# Redox metabolism in cell senescence: focusing on contributions from the metabolomic field

**DOI:** 10.3389/fmolb.2025.1754469

**Published:** 2026-01-09

**Authors:** Eliana Chacón, Guillermo Grünwaldt, Inés Marmisolle, Jennyfer Martínez, Celia Quijano

**Affiliations:** Departamento de Bioquímica, Facultad de Medicina, and Centro de Investigaciones Biomédicas (CEINBIO), Universidad de la República, Montevideo, Uruguay

**Keywords:** glutathione, metabolism, metabolomics, redox homeostasis, senescence

## Abstract

Cell senescence is triggered by stressful stimuli, including telomere attrition, genotoxic agents, and strong mitogenic signals. This state is characterized by proliferation arrest and acquisition of a senescence-associated secretory phenotype. Senescent cells secrete growth factors, chemokines, cytokines, proteases, and other factors that can impact the cell’s microenvironment, promoting aging and the development of age-associated diseases. These discoveries have emphasized the need for a detailed analysis of the senescent phenotype. Redox alterations are one of the hallmarks of cellular senescence, and are required to maintain the senescent phenotype. Here, we review current information on senescent cell’s redox metabolism, with a special focus on metabolomic profiling of human fibroblasts. We describe metabolic pathways involved in redox homeostasis, in particular glutathione metabolism, that undergo reprogramming in cell senescence, and links with the senescent phenotype.

## 1 Introduction

Hayflick and Moorhead first described cellular senescence as a limit to cellular proliferation due to progressive telomere shortening in human fibroblasts (Hayflick, 1965). It is now recognized as a broader stress response pathway that occurs *in vivo* (Collado and Serrano, 2010; Suryadevara et al., 2024). Senescence is characterized by a stable cell cycle arrest and multiple phenotypic changes, including the secretion of a wide array of pro-inflammatory cytokines, proteases, and other factors collectively known as the senescence-associated secretory phenotype (SASP) (Coppe et al., 2008).

As several of the changes associated with senescence can also be observed in other cellular states, proper identification of cellular senescence requires the presence of multiple hallmarks. Among them are: cell cycle arrest (mediated by p53/p21/pRb and/or p16/pRb), activation of the DNA damage response (DDR), heterochromatin changes, upregulation of anti-apoptotic pathways, acquisition of the SASP, increased lysosomal content, metabolic adaptations, cell surface markers, and morphological alterations (Suryadevara et al., 2024; Campisi, 2013).

The biological roles of senescent cells are complex and likely not yet fully understood. Physiological, beneficial functions have been described in oncogene-expressing cells, where senescence acts as a tumor-suppressive mechanism, in wound healing (Demaria et al., 2014), and during embryonic development, contributing to proper tissue formation (Muñoz-Espín et al., 2013; Storer et al., 2013). In other contexts, however, the accumulation of non-proliferating senescent cells and prolonged SASP exposure have detrimental effects, being associated with chronic inflammation, tissue dysfunction, and active contribution to the ageing process (Baker et al., 2011; Baker et al., 2016).

As research on cellular senescence progresses, it has become increasingly evident that senescence is not a singular, uniform state but rather encompasses a spectrum of phenotypes and cellular programs. These divergent states are shaped by multiple factors, including the nature of the inducing stimulus, the cell type involved, and the surrounding physiological context (Hernandez-Segura et al., 2017; Kirschner et al., 2020; Marmisolle et al., 2025).

Metabolomics has emerged more recently as a powerful tool among the various omics approaches increasingly used in cell research. Its ability to reflect upstream cellular characteristics—such as gene expression and protein function—while also providing a sensitive readout of external stimuli and niche-dependent interactions, makes it particularly well-suited to capture the dynamic and context-dependent nature of cell states (Bispo et al., 2021). Given the complexity, heterogeneity, and environmental responsiveness observed in senescent cells, these features underscore the potential of metabolomics to advance our understanding of cellular senescence. Among the hallmarks of senescence we find metabolic alterations, including an increase in reactive oxygen species formation at the mitochondrial level (Suryadevara et al., 2024), that participates in the induction and maintenance of the senescent phenotype (Lee et al., 1999; Correia-Melo et al., 2014; Passos et al., 2010; Borodkina et al., 2014).

In this review, we will examine current knowledge on redox metabolic changes associated with cellular senescence, with particular emphasis on studies that report intracellular metabolic profiling of human fibroblasts. We will describe some key redox metabolic pathways that undergo reprogramming during senescence, in particular glutathione (GSH) metabolism, and discuss their potential impact on key features of the senescent phenotype, especially the SASP.

## 2 Redox metabolism

Redox metabolism is defined by the finely regulated network of reduction and oxidation reactions that can occur within a cell (Lennicke and Cochemé, 2021). It is closely linked to energy metabolism, and the production of reactive oxygen species (ROS) that results from electron transfer reactions (Lennicke and Cochemé, 2021; Quijano et al., 2016). ROS is a generic term for a wide variety of molecules derived from oxygen; including superoxide anion (O_2_^.-^), hydrogen peroxide (H_2_O_2_), hydroxyl radical (^.^OH), hypochlorous acid (HOCl), electrophiles derived from lipid oxidation such as 4-hydroxynonenal (HNE), and peroxynitrite (ONOO^−^) formed from O_2_^.-^ and nitric oxide (^.^NO) (Sies et al., 2022; Murphy et al., 2022). Peroxynitrite is also considered a reactive nitrogen species (RNS). ROS can undergo redox reactions that produce oxidative modifications in macromolecules, and as a consequence, alter the biological functions of a cell (Lennicke and Cochemé, 2021; Sies et al., 2022).

Indeed, enhanced levels of oxidant species within a cell can lead to permanent cellular damage and contribute to cell death (Redza-Dutordoir and Averill-Bates, 2016). More interesting, however, is the fact that ROS have been recognized as signaling molecules that can regulate enzyme activity, transcription factors, and epigenetic modifications, affecting gene expression and cell function (Lennicke and Cochemé, 2021; Holmström and Finkel, 2014; Finkel, 2003). Antioxidant systems play a key role in maintaining ROS at low steady-state levels required for signaling. These include both enzymes, such as superoxide dismutase (SOD), catalase, glutathione peroxidase, glutathione reductase, thioredoxins, peroxiredoxins, and non-enzymatic antioxidants such as glutathione, vitamin C, and E (Quijano et al., 2016; Jena et al., 2023). Mantaining redox homeostasis – the balance between oxidants and antioxidants in cells – is key for cellular physiology, and its alteration impacts on cellular processes, drives cellular responses, and is implicated in the development of several diseases (Lennicke and Cochemé, 2021).

### 2.1 Glutathione metabolism

The tripeptide glutathione (L-γ-glutamyl-L-cysteinyl-glycine) is the predominant low molecular weight thiol present in cells (Tossouni et al., 2024). Inside cells, glutathione’s cysteine residue is mainly found in its reduced form as thiol (GSH) (>98%); however, it can be oxidzed by two electrons forming glutathione disulfide (GSSG) (Lu, 2013). Glutathione is required for iron–sulfur cluster biogenesis, cysteine storage, xenobiotic detoxification, and maintenance of redox homeostasis (Bachhawat and Yadav, 2018).

**FIGURE 1:**
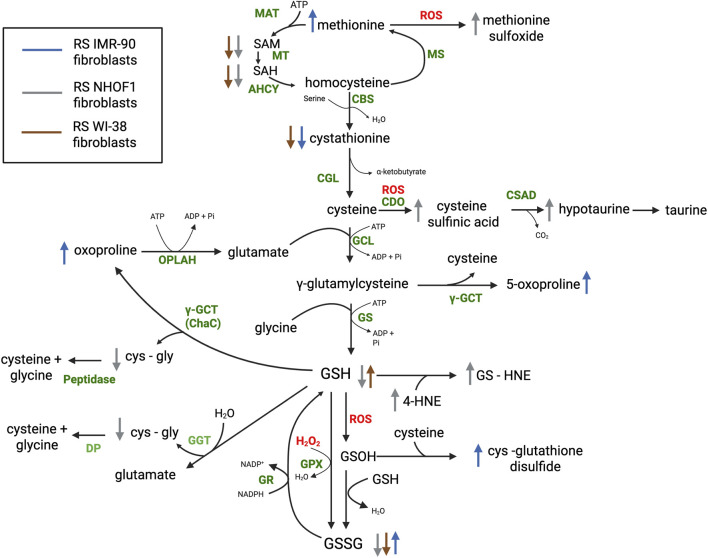
Intracellular metabolites involved in redox homeostasis pathways in replicative senescent fibroblasts. Schematic representation of metabolic pathways involved in glutathione, cysteine and methionine metabolism in human fibroblasts undergoing replicative senescence vs. non-senescent cells (confluent or quiescent). The arrows represent fold-increase (>1) or -decrease (<1) in concentration of intracellular metabolites with significant changes between conditions. Data and references can be found in Table 1. Reactive oxygen species are shown in red; and enzymes in green. Abbreviations: AHCY, adenosylhomocysteinase; CBS, cystathionine‐β‐synthase; CDO, cysteine dioxygenase; CGL, cystathionine‐γ‐lyase; CSAD, cysteinesulfinate decarboxylase; GCL, glutamate-cysteine ligase; γ-GCT, γ-glutamyl cyclotransferase; GPX, glutathione peroxidase; GR, glutathione reductase; GS, glutathione synthetase; GSH, reduced glutathione; GSSG, glutathione disufide; GS-HNE, 4-hydroxy-nonenal-glutathione; MAT, methionine adenosyltransferase; MS; MT, methyltransferases; OPLAH, 5-oxoprolinase; SAH, S-adenosylhomocysteine; SAM, S-adenosylmethionine. Created in BioRender. Quijano, C. (2026) https://BioRender.com/pbkbpr6.

GSH has a high concentration (1–15 mM) at the intracellular level, when compared to other antioxidant molecules (Lu, 2013; Deponte, 2017), and it can scavenge radical and non-radical oxidant species directly and indirectly through enzymatic reactions (Wu et al., 2004). Glutathione peroxidase (GPX) catalyzes the reduction of hydrogen peroxide and lipid peroxides by GSH, forming GSSG. There are several isoforms of GPX, with GPX4 playing a key role in the protection from ferroptosis, an iron-dependent form of necrosis, highlighting the protective role of GSH (Feng et al., 2023). GSSG can then be reduced back to GSH by NADPH in a reaction catalyzed by glutathione reductase (GR) (Figure 1) (Lu, 2013; Flohé et al., 2022). The GSH/GSSG redox couple is considered the main cellular redox buffer and, as such, the concentration ratio between reduced and oxidized forms of the tripeptide ([GSH]/[GSSG] ratio) is often used as an indicator of the cellular redox state (Wu et al., 2004).

GSH cysteine residue can also be oxidized directly, in a non-catalyzed reaction, by several oxidants such as hydrogen peroxide, peroxynitrite, or hypochlorous acid, forming glutathione sulfenic acid (GSOH), which can in turn react with a second GSH molecule forming GSSG (Figure 1). Alternatively, GSOH may undergo further oxidation to sulfinic (GSO_2_H) or sulfonic acid (GSO_3_H) (Gupta and Carroll, 2014). GSOH can also react with other thiols, such as cysteine residues, forming mixed disulfides (e.g., cystein-glutathione disulfide) (Gupta and Carroll, 2014). This can occur with cysteine residues in proteins, and the process is termed glutathionylation. GSH also reacts with lipid oxidation products, such as the highly electrophilic 4-hydroxy-2-nonenal (HNE), producing GS-HNE adducts (Stadtman and Levine, 2000; Figure 1). Therefore, glutathione metabolism is aimed at maintaining harmful ROS, RNS and, GSSG at low levels and GSH at high levels to maintain redox homeostasis. In response to oxidative stress, cellular GSH levels decrease significantly, and the oxidized derivatives of this molecule increase (Wu et al., 2004).

Glutathione synthesis starts with the reaction between cysteine and glutamate to form γ-glutamyl-cysteine. The reaction consumes ATP and is catalyzed by glutamate-cysteine ligase (GCL). Glycine incorporation also requires ATP and is catalyzed by GSH synthetase (GS) (Figure 1). The first step, catalyzed by glutamate-cysteine ligase (GCL), is rate-limiting and regulated by GSH (competitive inhibitor) and by cysteine availability (Lu, 2013). Therefore, cysteine metabolism is essential for GSH synthesis, and will be analyzed later.

GSH degradation is catalized by γ-glutamyltranspeptidase (GGT), an enzyme that catalyzes the cleavage of the peptide bond between cysteine and the γ-carboxyl group of glutamate, and the transfer of the γ-glutamyl moiety to water (hydrolysis), an amino acid, or a short peptide (transpeptidation) (Mitrić and Castellano, 2023). This enzyme is present on the outer membrane of cells, and the active site faces the extracellular space; thus, GSH can be metabolized extracellularly (Lu, 2013). The transfer of the γ-glutamyl group to water renders glutamate, while its coupling to an amino acid can form a γ-glutamyl amino acid (GGAA) (Mitrić and Castellano, 2023). However, since the Km for water is lower (μM) than the Km for amino acids (mM) and physiological levels of amino acids in the extracellular space are in the μM range, the hydrolysis reaction predominates (Bachhawat and Yadav, 2018; Mitrić and Castellano, 2023) (Figure 1). The hydrolysis of the peptide bond in GSH, catalyzed by GGT, releases cysteinyl-glycine that can be cleaved by cell surface dipeptidases (DP) into cysteine and glycine, that are transported back into the cell (Lu, 2013). Another pathway for GSH degradation at the intracellular level is the reaction catalyzed by glutathione specific γ-glutamyl cyclotransferases 1 and 2 (CHAC1/2). This reaction yields 5-oxoproline (pyroglutamic acid) while releasing cysteinyl-glycine (Bachhawat and Yadav, 2018). Cysteinyl-glycine can be hydrolyzed by a peptidase, while 5-oxoproline is cleaved by 5-oxoprolinase (OPLAH) to yield glutamate (Bachhawat and Yadav, 2018). Oxoproline can also be formed from γ-glutamyl-cysteine in a reaction catalyzed γ-glutamyl cyclotransferase (γ-GCT) (Figure 1; Bachhawat and Yadav, 2018).

### 2.2 Cysteine and methionine metabolism

Cysteine can be obtained from the diet or protein breakdown, but it can also be synthesized from methionine in the liver. This pathway begins with S-adenosylmethionine formation (SAM) from methionine and ATP in a reaction catalyzed by methionine adenosyltransferase (MAT) (Parkhitko et al., 2019). SAM then donates a methyl group to several acceptor molecules (e.g., DNA, RNA, proteins, metabolites), generating S-adenosylhomocysteine (SAH), which is then hydrolyzed to homocysteine and adenosine, in reactions catalyzed by methyltransferases (MT) and adenosylhomocysteinase (AHCY, SAH hydrolase), respectively (Figure 1; Parkhitko et al., 2019). These methylation reactions are involved in epigenetic regulation of gene expression (Ducker and Rabinowitz, 2017). Homocysteine can be remethylated to form methionine and retained in the methylation cycle, or converted to cysteine in the transsulfuration pathway (Parkhitko et al., 2019). In this pathway, homocysteine condenses with serine to form cystathionine, and this reaction, catalyzed by cystathionine‐β‐synthase (CBS), is the rate‐limiting step of the route. Cystathionine is then hydrolyzed by cystathionine‐γ‐lyase (CGL) to produce cysteine and α-ketobutyrate (2-oxobutyrate) (Figure 1; Parkhitko et al., 2019). Ophtalmate, a product of α-ketobutyrate metabolism, regulates transporters and enzymes involved in GSH uptake, efflux, and metabolism (Schomakers et al., 2024).

Cysteine concentrations are tightly regulated; in particular by cysteine dioxygenase (CDO) which catalyzes the oxygenation of the sulfur atom, rendering cysteine sulfinic acid, which is then degraded to hypotaurine and taurine, preventing its accumulation (Stipanuk et al., 2009). Hypotaurine is formed by the decarboxylation of cysteine sulfinic acid in a reaction catalyzed by cysteinesulfinate decarboxylase (CSAD), and afterwards is oxidized to taurine (Stipanuk, 2004). Overoxidation of cysteine by ROS or RNS can also produce cysteine sulfinic acid, as well as other oxidized intermediates (Hayward and Baud, 2025; Figure 1).

## 3 Redox metabolism in senescent cells

In senescent cells, there is a clear disruption of redox homeostasis, with a shift towards a more oxidative environment. ROS levels are increased and drive the acquisition and/or maintenance of the senescent phenotype (Lee et al., 1999; Correia-Melo et al., 2014; Takahashi et al., 2006; NYl et al., 2021). ROS are formed in mitochondrial catabolic energy-producing pathways, at the electron transport chain complexes, and by enzymes with flavin cofactors (Lee et al., 1999; Quijano et al., 2016; Moiseeva et al., 2009; Passos et al., 2007; Kowaltowski et al., 2009). In addition, NADPH oxidase 4 (NOX4), has been identified as a source of oxidant species in senescence (Weyemi et al., 2012; Lener et al., 2009; Sakai et al., 2018).

DNA oxidation can trigger the activation of the DDR, an essential mechanism for the induction and establishment of senescence (Passos et al., 2010; Borodkina et al., 2014; d’Adda di Fagagna, 2008). Likewise, proteomic approaches have shown evidence of protein oxidation in senescent cells; numerous proteins have been found carbonylated or forming adducts with 4-hydroxynonenal (HNE) (Stadtman and Levine, 2000). Furthermore, ROS can oxidize cysteine, methionine, tyrosine, tryptophan, histidine, leucine, valine, lysine, and arginine residues in proteins (Stadtman and Levine, 2000). Among them, protein cysteine oxidation appears as a key post-translational modification, involved in signaling events (Holmström and Finkel, 2014). It is interesting to mention that several oxidatively modified proteins have been identified in organ aging and age-related diseases, highlighting the importance of redox metabolism (Stadtman and Levine, 2000; Baraibar et al., 2012). Moreover, in a recent report, Xiao et al. identified relevant changes in protein cysteine oxidation in aged mice, defining new mechanisms of redox regulation in aging (Xiao et al., 2020).

Senescent cells also present changes in antioxidant systems, that contribute to the alteration in redox homeostasis, and metabolomic studies have provided interesting insights into glutathione metabolism in this setting. Both decreases and increases in reduced glutathione (GSH) and glutathione disulfide (GSSG) levels were reported in fibroblasts undergoing senescence, including replicative senescence (RS), oncogene-induced senescence (OIS), and therapy-induced senescence (TIS) (Quijano et al., 2012; James et al., 2016; Tighanimine et al., 2024) (Table 1; Figure 1; Supplementary Table S1). However, oxidized forms of glutathione, such as cysteine-glutathione disulfide, and 4-hydroxy-nonenal-glutathione (GS-HNE), were higher in senescent than control fibroblasts both in replicative and oncogene-induced senescence (Quijano et al., 2012; James et al., 2016) (Table 1; Figure 1; Supplementary Table S1). As we mentioned above, methionine and cysteine are susceptible to oxidation, forming methionine sulfoxide, cysteine–glutathione disulfide, and cysteine sulfinic acid, all of which were higher in fibroblasts undergoing replicative senescence than in controls (Quijano et al., 2012; James et al., 2016; Piro et al., 2024; James et al., 2015) (Table 1; Figure 1). Hypotaurine, a metabolite derived from cysteine sulfinic acid metabolism, was also increased in replicative senescent fibroblasts, with respect to control cells (James et al., 2016; Tighanimine et al., 2024; James et al., 2015), but not in mesenchymal stromal cells (MSC) or endothelial cells (Supplementary Table S1); nor in OIS fibroblasts (Quijano et al., 2012).

**TABLE 1:** Intracellular metabolites involved in redox homeostasis pathways in replicative senescent fibroblast**s**.

Pathway	Metabolite	Fold change Sen/Non-Sen	Cell type	Inducing stimuli	Reference
GSH metabolism	GSH	2.5	fibroblasts	RS/Q	Tighanimine et al. (2024)
	GSH	<1	fibroblasts	RS/Q	James et al. (2015)
	GSSG	1.99	fibroblasts	RS/Conf	Quijano et al. (2012)
	GSSG	<1	fibroblasts	RS/Q	James et al. (2016)
	GSSG	0.1	fibroblasts	RS/Q	Tighanimine et al. (2024)
	cysteine-glutathione disulfide	12.93	fibroblasts	RS/Conf	Quijano et al. (2012)
	cysteinyl-glycine	<1	fibroblasts	RS/Q	James et al. (2016)
	γ-glutamyl-isoleucine	0.66	fibroblasts	RS/Conf	Quijano et al. (2012)
	γ-glutamyl-methionine	3.81	fibroblasts	RS/Conf	Quijano et al. (2012)
	γ-glutamyl-glutamate	<1	fibroblasts	RS/Q	James et al. (2016)
	γ-glutamyl-glutamine	7.73	fibroblasts	RS/Conf	Quijano et al. (2012)
	γ-glutamyl-glutamine	<1	fibroblasts	RS/Q	James et al. (2015)
	γ-glutamyl-phenylalanine	2.80	fibroblasts	RS/Conf	Quijano et al. (2012)
	γ-glutamyl-phenylalanine	>1	fibroblasts	RS/Q	James et al. (2016)
	γ-glutamyl-phenylalanine	>1	fibroblasts	RS/Q	James et al. (2015)
	γ-glutamyl-alanine	>1	fibroblasts	RS/Q	James et al. (2016); James et al. (2015)
	oxoproline	1.57	fibroblasts	RS/Conf	Quijano et al. (2012)
	S-lactoylglutathione	>1	fibroblasts	RS/Q	James et al. (2016)
	4-hydroxy-nonenal-glutathione (GS-HNE)	>1	fibroblasts	RS/Q	James et al. (2016)
Cysteine/Methionine metabolism	cystathionine	0.28	fibroblasts	RS/Conf	Quijano et al. (2012)
	cystathionine	0.5	fibroblasts	RS/Q	Tighanimine et al. (2024)
	ophtalmate	0.42	fibroblasts	RS/Conf	Quijano et al. (2012)
	ophtalmate	>1	fibroblasts	RS/Q	James et al. (2016)
	cysteine sulfinic acid	>1	fibroblasts	RS/Conf	James et al. (2015)
	hypotaurine	>1	fibroblasts	RS/Q	James et al. (2016)
	methionine	1.31	fibroblasts	RS/Conf	Quijano et al. (2012)
	methionine sulfoxide	>1	fibroblasts	RS/Q	James et al. (2016)
	S-adenosylhomocysteine (SAH)	0.8#	fibroblasts	RS/Q	Tighanimine et al. (2024)
	S-adenosylhomocysteine (SAH)	<1	fibroblasts	RS/Conf	James et al. (2016)
	S-adenosylmethionine (SAM)	<1	fibroblasts	RS/Q	James et al. (2016)
	S-adenosylmethionine (SAM)	0.5	fibroblasts	RS/Q	Tighanimine et al. (2024)

Metabolomic studies were analyzed, and fold-change in intracellular metabolites involved in glutathione, cysteine and methionine metabolism were obtained for human fibroblasts undergoing replicative senescence (Sen) vs. non-senescent cells (Non-sen, confluent or quiescent). Fold-increase (>1) or -decrease (<1) in concentration of intracellular metabolites with significant changes between contitions is shown (P < 0.05 or #0.1 > P > 0.05). Different senescence-inducing stimuli were considered: (i) Human lung fibroblasts (IMR-90) undergoing replicative senescence (RS) vs. confluent cells (Conf) (Quijano et al., 2012). (ii) Human lung fibroblasts (WI-38) undergoing replicative senescence (RS) vs. quiescent cells (Q) (Tighanimine et al., 2024). (iii) Human oral fibroblasts (NHOF-1) undergoing replicative senescence (RS) vs. quiescent (Q) or confluent cells (Conf) (James et al., 2016; James et al., 2015). Comparisons with confluent or quiescent cells, instead of proliferating cells, were made to focus on differences that were not due to proliferation arrest in senescent cells.

Additionally, SAM, SAH, cystathionine, and γ-glutamyl-cysteine, metabolites involved in cysteine and GSH biosynthesis, were decreased in fibroblasts undergoing replicative senescence or oncogene-induced senescence (Quijano et al., 2012; James et al., 2016; Tighanimine et al., 2024); as well as in therapy-induced senescent breast cancer cells, with respect to non-senescent cells (Wu et al., 2017) (Table 1; Figure 1; Supplementary Table S1). On the other hand, high levels of oxoproline were found in fibroblasts, MSC, and keratinocytes undergoing replicative senescence, pointing to an increase in GSH degradation (Quijano et al., 2012; Piro et al., 2024; Fernandez-Rebollo et al., 2020) (Table 1; Figure 1). In addition, changes in expression of enzymes that consume GSH, such as glutathione peroxidase (GPX1), γ-glutamyltranspeptidase (GGT) and glutathione S-transferase P (GSTP1, involved in detoxification of hydrophobic molecules), were observed in replicative senescent keratinocytes (Piro et al., 2024). Together, these results suggest GSH homeostasis is compromised in senescent cells, by a decrease in synthesis, an increase in degradation, and/or reaction with oxidative species. Changes in these pathways probably contribute to the redox imbalance that occurs in senescent cells.

Interestingly, metabolomics data showed increased levels of intracellular GGAAs in OIS, and RS in fibroblasts and keratinocytes (Table 1; Supplementary Table S1). These include γ-glutamyl-leucine, γ-glutamyl-methionine, γ-glutamyl-phenylalanine and γ-glutamyl-tyrosine (Quijano et al., 2012; James et al., 2016; Piro et al., 2024; James et al., 2015). Until recently, GGAAs were considered important intermediates in GSH breakdown, formed in transpeptidation reactions catalyzed by extracellular GGT. However, as described before, this reaction does not seem to be physiologically relevant (Bachhawat and Yadav, 2018). Instead, recent reports indicate these peptides could be synthesized in a reaction catalyzed by GCL when cysteine is lacking, since this enzyme has broad substrate specificity (Ikeda and Fujii, 2023; Kang et al., 2021). In fact, γ-glutamyl-cysteine was reduced in OIS, supporting a role for GCL in GGAA synthesis (Quijano et al., 2012).

The role of these dipeptides is not clear yet, but GGAAs synthesis has been described to prevent ferroptosis upon cysteine deprivation (Kang et al., 2021), and extracellular GGAAs can modulate the activty of calcium-sensing receptors (CaSRs) (Ikeda and Fujii, 2023). Therefore γ-glutamyl peptides may constitute a new class of signaling molecules.

## 4 Redox regulation of the senescent phenotype

As we mentioned before, several lines of evidence show that redox metabolism plays an important role in the induction and maintenance of the senescent phenotype, through different signaling pathways (Lee et al., 1999; Correia-Melo et al., 2014; Takahashi et al., 2006; NYl et al., 2021).

In first place, it has been proved that oxidant species activate, and maintain the persistent activation of the DDR, during senescence (Passos et al., 2010). Oxidant species can cause double-strand breaks in the DNA, which in turn activate ATM kinase, that promotes the stabilization of tumor suppressor p53 which increases the expression of cyclin-dependent kinase inhibitor 1 (*CDKN1A*, p21). P53 and p21 are then capable of contributing to the increase in intracellular ROS during senescence, providing a positive feedback loop for the maintenance of the senescence phenotype (Passos et al., 2010; Vigneron and Vousden, 2010; Rodier et al., 2009; Macip et al., 2002). In addition, senescent cells can also activate the DDR in neighbouring cells via gap junction-mediated cell-cell contact in a process that involves oxidants, resulting in the induction of what is known as secondary senescence (Nelson et al., 2012). Finally, it has been shown that persistent DNA damage signaling contributes to the secretion of the SASP factors, highlighting the role of oxidants in senescent cells and their impact on the organism (Rodier et al., 2009).

Mitogen-activated protein kinase p38 (p38 MAPK) also participates in the induction of the SASP, but this occurs independently of the DDR pathway (Freund et al., 2011). p38 MAPK is activated by several senescence-inducing stimuli and can promote nuclear translocation of nuclear factor kappa B (NF-κB) which results in the induction of the SASP (Freund et al., 2011; Wang et al., 2024). Interestingly, a positive feedback loop could also be established between p38 MAPK and oxidant species, since this kinase can promote the increase in ROS levels (Borodkina et al., 2014), and oxidant species have been shown to activate p38 MAPK in senescent cells (Marmisolle et al., 2017).

Another relevant pathway involved in the regulation of the SASP is the cyclic GMP–AMP synthase–stimulator of interferon genes (cGAS–STING) pathway. In senescent cells, cytosolic chromatin fragments and mtDNA, released from mitochondria, act as damage-associated molecular patterns (DAMPs), that are recognized by cGAS (Victorelli et al., 2023; Dou et al., 2017; Glück et al., 2017). Upon binding to double-stranded DNA, cGAS catalyzes the synthesis of cyclic dinucleotide GMP-AMP (cGAMP) that binds to STING, which in turn recruits tank-binding kinase 1 that activates interferon regulatory factor 3, resulting in type I IFN production, as well as NF-κB, leading to the expression of proinflammatory cytokines (Victorelli et al., 2023; Dou et al., 2017; Glück et al., 2017). ROS can play an indirect but important role in the activation of the cGAS–STING pathway. On one side, oxidized mtDNA can be cleaved by the Flap endonuclease 1 (FEN1) to 500–650 bp fragments, and exit mitochondria leading to the activation of the pathway (Xian and Karin, 2023; Xian et al., 2022). Further more, oxidized DNA is more resistant to degradation by cytosolic nucleases (Gehrke et al., 2013), therefore oxidants can promote the accumulation of DNA in the cytosol, and potentiate the activation of the cGAS-STING pathway (Gehrke et al., 2013).

In agreement with the described role for oxidants in senescence, peroxiredoxins (PRX), which are well-known antioxidant enzymes, have been shown to protect cells from senescence and to participate in the SASP. Loss of PRX1 in mouse embryonic fibroblasts led to the accumulation of ROS, increased p16 expression and senescence-associated β-galactosidase activity, classical markers of senescence (Park et al., 2017). While in human retinal pigment epithelial cells, where senescence was induced by exposure to radiation, PRX6 silencing decreased the expression and secretion of several SASP components, including IL-6 (Salovska et al., 2022).

Antioxidant strategies have been valuable tools to understand the role of oxidant species in senescence, among them is N-acetylcysteine (NAC), a cysteine prodrug that replenishes intracellular GSH level. NAC increases the availability of cysteine, the limiting substrate for GSH synthesis; and, by doing so, it reinforces the antioxidant capacity of a cell and decreases the level of oxidant species (Atkuri et al., 2007). The Polycomb complex protein Bmi1, is a repressor of the Ink4a/Arf locus that encodes p16 and p14/p19 (Park et al., 2004; Itahana et al., 2003), implicated in cell cycle regulation and senescence. In mice deficient in Bmi1, NAC reduced ROS levels and DDR activation in thymocytes, rescued thymic developmental defects and animals failure to thrive (Liu et al., 2009). In a more recent study, several markers of oxidative stress (8-hydroxy-2′-deoxyguanosine), senescence, and pulmonary fibrosis were found in the lungs of Bmi1 knockout animals with respect to controls. NAC treatment resulted in a decrease in all of these markers, ameliorated pulmonary dysfunction and prolonged lifespan in Bmi1 deficient mice (Chen et al., 2020). These results underscore, redox metabolism in senescence as a research area with actual therapeutic potential, and metabolomics as a powerful tool to move forwards in this field.

## 5 Conclusion

In sum, metabolomic data presented herein support that senescent cells undergo changes in redox homeostasis, including a decrease in GSH synthesis, an increase in GSH degradation and reaction with oxidative species. Comparison of metabolites in senescent vs. non-senescent, yielded different results depending on the senescent inducing stimuli and cell type, as has been described for gene expression and bioenergetics (Table 1) (Hernandez-Segura et al., 2017; Marmisolle et al., 2025). Its worth noting that the proliferation status of non-senescent cells (quiescent/confluent or proliferating) also affected the metabolite ratio between senescent/non-senescent cells (Table 1; Supplementary Table S1).

Though this approach, based on the analysis of metabolic profiles, resulted useful and revealing, we must acknowledge its limitations. In particular, studies used for this review (Quijano et al., 2012; James et al., 2016; Tighanimine et al., 2024; Piro et al., 2024; James et al., 2015; Wu et al., 2017; Fernandez-Rebollo et al., 2020; Ya et al., 2024) were performed measuring intracellular steady-state concentrations of metabolites in cells cultured in media with high concentrations of nutrients, including glutamine a precursor of glutamate a GSH component. Future metabolomic studies on senescent cells performed *in vivo,* or in culture but with a more controled environment, with physiological concentrations of nutrients, would allow a more accurate description of redox metabolism in senescence (Jang et al., 2018). Additionally isotope-assisted metabolic flux analysis, would improve our understanding of pathway activities (Jang et al., 2018).
