# Supplementary material for: Redox metabolism in cell senescence: focusing on contributions from the metabolomic field
Source: Front Mol Biosci. 2026 Jan 9;12:1754469. doi: 10.3389/fmolb.2025.1754469 (PMC12853661; doi:10.3389/fmolb.2025.1754469)
Supplement: Supplementary file 1 [file DataSheet1.pdf]

Redox metabolism in cell senescence: focusing on contributions from the metabolomic field

Eliana Chacón[1], Guillermo Grünwaldt[1], Inés Marmisolle[1], Jennyfer Martínez[1], Celia Quijano[1*].

[1]Departamento de Bioquímica, Facultad de Medicina, and Centro de Investigaciones Biomédicas (CEINBIO), Universidad de la República, Montevideo, Uruguay.

*** Correspondence:** Celia Quijano
celiq@fmed.edu.uy; celia.quijano@gmail.com

Table 1: Intracellular metabolites involved in redox homeostasis pathways in senescent cells. Metabolomic studies were analyzed, and fold-change in metabolites involved in glutathione, cysteine and methionine metabolism were obtained for senescent vs. non-senescent cells. Fold-increase (>1) or -decrease (<1) in the concentration of intracellular metabolites with significant changes in concentration are shown (P<0.05, #0.5<P<0.1). Different cell types and senescence-inducing stimuli were considered:

1. Human lung fibroblasts (IMR-90) expressing H-RAS^{G12V} (OIS RAS) vs. confluent cells (Conf) (1).
2. Human lung fibroblasts (WI-38) expressing H-RAS^{G12V}(OIS RAS) vs. proliferating cells (P) (2).
3. Human lung fibroblasts (WI-38) expressing BRAF^{V600E} (OIS RAF) vs. proliferating cells (P) (2).
4. Human lung fibroblasts (WI-38) undergoing replicative senescence (RS) vs. proliferating cells (P) (2).
5. Human lung fibroblasts (WI-38) exposed to etoposide (TIS) vs. proliferating cells (P) (2).
6. Human oral fibroblasts (NHOF-1) undergoing replicative senescence (RS) vs. quiescent (Q) or confluent cells (Conf) (3,4).
7. Human breast cancer cells (MCF7) exposed to doxorubicin (TIS) vs. exposed to DMSO (Ctrl) (5).
8. Human mesenchymal stromal cells (MSC) undergoing replicative senescence (RS) vs. control cells (Ctrl) (6).
9. Human epidermal keratinocytes (NHEK) undergoing replicative senescence (RS) vs. control (Ctrl) cells (7).

11. Human brain microvascular endothelial cells (HBMECs) undergoing replicative senescence vs. confluent cells (Conf) (8).

**Pathway**	**Metabolite**	**Fold change Sen/Ctrl**	**Cell Type**	**Inducing stimuli**	**Ref**
GSH metabolism	GSH	0.67	fibroblasts	OIS RAS/Conf	(1)
	GSH	0.7	fibroblasts	OIS RAS/P	(2)
	GSH	2.8	fibroblasts	RS/P	(2)
	GSH	<1#	fibroblasts	RS/P	(3)
	GSH	0.4	fibroblasts	OIS RAF/P	(2)
	GSH	2	fibroblasts	TIS/P	(2)
	GSH	0.68	Breast cancer	TIS/Ctrl	(5)
	GSSG	0.49	fibroblasts	OIS/P	(2)
	GSSG	<1	fibroblasts	RS/P	(4)
	GSSG	3.9	fibroblasts	TIS/P	(2)
	GSSG	1.76	Breast cancer	TIS/Ctrl	(5)
	GSSG	3.9	fibroblasts	TIS/P	(2)
	cysteine-glutathione disulfide	5.56	fibroblasts	OIS RAS/Conf	(1)
	cysteine-glutathione disulfide	0.5	MSC	RS/Ctrl	(6)
	glutamate	0.83	fibroblasts	OIS RAS/Conf	(1)
	glycine	1.39	fibroblasts	OIS RAS/Conf	(1)
	glycine	0.39	fibroblasts	OIS RAF/P	(2)
	glycine	2.1	fibroblasts	TIS/P	(2)
	cysteinyl-glycine	<1	fibroblasts	RS/P	(4)
	γ-glutamyl-cysteine	0.60	fibroblasts	OIS RAS/Conf	(1)
	γ-glutamyl-valine	1.44	fibroblasts	OIS RAS/Conf	(1)
	γ-glutamyl-leucine	2.7	fibroblasts	OIS RAS/Conf	(1)
	γ-glutamyl-leucine	1.41	keratinocytes	RS/Ctrl	(7)
	γ-glutamyl-isoleucine	2.32	fibroblasts	OIS RAS/Conf	(1)
	γ-glutamyl-methionine	2.97	fibroblasts	OIS RAS/Conf	(1)
	γ-glutamyl-methionine	1.51	keratinocytes	RS/Ctrl	(7)
	γ-glutamyl-glutamate	1.58	fibroblasts	OIS RAS/Conf	(1)
	γ-glutamyl-glutamate	<1	fibroblasts	RS/P	(4)
	γ-glutamyl-glutamine	1.34	fibroblasts	OIS RAS/Conf	(1)
	γ-glutamyl-glutamine	<1	fibroblasts	RS/P	(3)
	γ-glutamyl-phenylalanine	3.06	fibroblasts	OIS RAS/Conf	(1)
	γ-glutamyl-phenylalanine	>1	fibroblasts	RS/Q	(4)
	γ-glutamyl-phenylalanine	4.01	MSC	RS/Ctrl	(6)
	γ-glutamyl-phenylalanine	1.50	keratinocytes	RS/Ctrl	(7)
	γ-glutamyl-tyrosine	3.79	fibroblasts	OIS RAS/Conf	(1)
	γ-glutamyl-tyrosine	1.58	keratinocytes	RS/Ctrl	(7)
	γ-glutamyl-alanine	>1	fibroblasts	RS	(4)
	oxoproline	0.64	fibroblasts	OIS RAS/Conf	(1)
	oxoproline	1.7	keratinocytes	RS/Ctrl	(7)
	oxoproline	2.6	MSC	RS/Ctrl	(6)

	S-lactoylglutathione	>1	fibroblasts	RS	(4)
	4-hydroxy-nonenal-glutathione (GS-HNE)	>1	fibroblasts	RS	(4)
Cys/Met metabolism	cysteine	3.46	fibroblasts	OIS RAS/Conf	(1)
	cystine	0.26	fibroblasts	TIS/P	(2)
	cystine	0.13	fibroblasts	RS/P	(2)
	homocysteine	1.46	Breast cancer	TIS/Ctrl	(5)
	cystathionine	0.5	fibroblasts	OIS RAS/P	(2)
	cystathionine	<1	fibroblasts	RS/P	(4)
	cystathionine	2.7	fibroblasts	RS/P	(2)
	cystathionine	0.41	Breast cancer	TIS/Ctrl	(5)
	ophtalmate	>1	fibroblasts	RS/P	(4)
	cysteine sulfinic acid	0.36	fibroblasts	OIS RAS/Conf	(1)
	cysteine sulfinic acid	>1	fibroblasts	RS/P	(4)
	hypotaurine	0.31	fibroblasts	OIS RAS/Conf	(1)
	hypotaurine	>1	fibroblasts	RS/P	(4)
	hypotaurine	0.21	MSC	RS/Ctrl	(6)
	hypotaurine	0.49	Endothelial cell	RS/Conf	(8)
	taurine	1.40	fibroblasts	OIS RAF/P	(2)
	taurine	2.1	fibroblasts	RS/P	(2)
	taurine	1.9	fibroblasts	TIS/P	(2)
	taurine	0.16	MSC	RS/Ctrl	(6)
	taurine	0.6	Endothelial cell	RS/Conf	(8)
	taurine	1.57	Breast cancer	TIS/Ctrl	(5)
	methionine	1.8	fibroblasts	OIS/P	(2)
	methionine	0.48	fibroblasts	OIS RAF/P	(2)
	methionine sulfoxide	>1	fibroblasts	RS/P	(4)
	methionine sulfoxide	2.14	keratinocytes	RS/Ctrl	(7)
	S-adenosylhomocysteine (SAH)	0.44	fibroblasts	OIS/P	(2)
	S-adenosylhomocysteine (SAH)	<1	fibroblasts	RS/P	(4)
	S-adenosylhomocysteine (SAH)	2.9	fibroblasts	RS/P	(2)
	S-adenosylhomocysteine (SAH)	4.4	fibroblasts	TIS/Ctrl	(2)
	S-adenosylmethionine (SAM)	0.79	fibroblasts	OIS RAS/P	(2)
	S-adenosylmethionine (SAM)	5.4	fibroblasts	RS/P	(2)
	S-adenosylmethionine (SAM)	<1	fibroblasts	RS/P	(4)
	S-adenosylmethionine (SAM)	1.6	fibroblasts	TIS/Ctrl	(2)

References

1. Quijano C, Cao L, Fergusson MM, Romero H, Liu J, Gutkind S, et al. Oncogene-induced senescence results in marked metabolic and bioenergetic alterations. Cell Cycle. 2012/03/17 ed. 2012 Apr 1;11(7):1383–92.

2. Tighanimine K, Nabuco Leva Ferreira Freitas JA, Nemazanyy I, Bankolé A, Benarroch-Popivker D, Brodesser S, et al. A homoeostatic switch causing glycerol-3-phosphate and phosphoethanolamine accumulation triggers senescence by rewiring lipid metabolism. Nat Metab. 2024 Feb 19;6(2):323–42.

3. James EL, Michalek RD, Pitiyage GN, de Castro AM, Vignola KS, Jones J, et al. Senescent human fibroblasts show increased glycolysis and redox homeostasis with extracellular metabolomes that overlap with those of irreparable DNA damage, aging, and disease. Journal of proteome research. 2015/02/19 ed. 2015 Apr;14(4):1854–71.

4. James EL, Lane JA, Michalek RD, Karoly ED, Parkinson EK. Replicatively senescent human fibroblasts reveal a distinct intracellular metabolic profile with alterations in NAD+ and nicotinamide metabolism. Scientific reports. 2016/12/08 ed. 2016 Dec;6:38489.

5. Wu M, Ye H, Shao C, Zheng X, Li Q, Wang L, et al. Metabolomics–Proteomics Combined Approach Identifies Differential Metabolism-Associated Molecular Events between Senescence and Apoptosis. J Proteome Res. 2017 June 2;16(6):2250–61.

6. Fernandez-Rebollo E, Franzen J, Goetzke R, Hollmann J, Ostrowska A, Oliverio M, et al. Senescence-Associated Metabolomic Phenotype in Primary and iPSC-Derived Mesenchymal Stromal Cells. Stem Cell Reports. 2020 Feb;14(2):201–9.

7. Piro MC, Pecorari R, Smirnov A, Cappello A, Foffi E, Lena AM, et al. p63 affects distinct metabolic pathways during keratinocyte senescence, evaluated by metabolomic profile and gene expression analysis. Cell Death Dis. 2024 Nov 14;15(11):830.

8. Ya J, Whitby A, Bayraktutan U. Metabolites and Metabolic Functional Changes—Potential Markers for Endothelial Cell Senescence. Biomolecules. 2024 Nov 20;14(11):1476.
